# The Association between Near Work Activities and Myopia in Children—A Systematic Review and Meta-Analysis

**DOI:** 10.1371/journal.pone.0140419

**Published:** 2015-10-20

**Authors:** Hsiu-Mei Huang, Dolly Shuo-Teh Chang, Pei-Chang Wu

**Affiliations:** 1 Department of Ophthalmology, Kaohsiung Chang Gung Memorial Hospital and Chang Gung University College of Medicine, Kaohsiung, Taiwan, R.O.C.; 2 Wilmer Eye Institute, Johns Hopkins University, Baltimore, Maryland, United States of America; The Chinese University of Hong Kong, HONG KONG

## Abstract

Myopia has a multifactorial etiology, although environmental factors are predominant in determining its current patterns. Currently, associations between near work activities and myopia have not been consistently observed. Therefore, we performed a systematic review to quantify the effect of near work activities on myopia in children. Relevant articles published between 1989 and 2014 were identified in MEDLINE, Embase, and the Cochrane Library, and the citation lists were reviewed. Twelve cohort studies and 15 cross-sectional studies were included (25,025 children aged between 6 and 18 years). The *I* ^2^ statistic was used to assess heterogeneity. Study-level data were pooled using a random-effects model or a fixed-effects model (when less than 5 studies were included). We found that more time spent on near work activities was associated with higher odds of myopia (odds ratio [OR] = 1.14; 95% confidence interval [CI] = 1.08–1.20) and that the odds of myopia increased by 2% (OR:1.02; 95% CI = 1.01–1.03) for every one diopter-hour (hr) more of near work per week. Therefore, the development of a strategy to reduce the impact of near work on myopia would be important for preventing myopia in children.

## Introduction

The prevalence of myopia has increased dramatically in recent years around the world and, in some highly educated groups such as law and medical students, it now exceeds 80% [1, 2]. Consistent with the increase in overall myopia, there has been an increase in the prevalence of high myopia. High myopia is an crucial public health problem because of its association with an increased risk of several ocular diseases including myopic retinal degeneration, retinal detachment, glaucoma, cataract, visual impairment, and blindness [3–5]. Therefore, it is important to identify the possible risk and preventive factors in the development of myopia.

Myopia is generally believed to have a multifactorial etiology [6], and early onset of myopia is associated with high myopia in adult life [7–9]. The rapid increase in the prevalence of myopia some area of the world suggests that environmental factors such as increasing educational pressures and urbanisation might be important factors in determining the current patterns of school myopia” [6, 10].

Near work is considered as the activities done at short working distance such as reading, studying (doing homework, writing), computer use/playing video games, or watching TV, etc.) [11–13]. Due to the high visual demands of near work including reading, and the tendency for myopia to develop during the school years, the time children spend engaging in reading and other near work has long been considered to be a potential cause to myopia development. However, the associations between time spent reading and myopia have not been consistently observed [12, 14–16]. Therefore, the aim of this review is to examine the magnitude of the association between time spent on near work and myopia by systematically identifying and quantitatively combining all available and relevant studies.

## Materials and Methods

### Search Strategy

We searched several databases (MEDLINE, Embase, and the Cochrane library), from April 1, 1989 to May 1, 2014, to identify potentially relevant articles. Our search included combined Medical Subject Headings and keywords for children with myopia (study population) and near work activities. The search terms included (“myopia” OR “short-sighted” OR “near-sighted” OR” refractive errors”) AND (“near work” OR “studying” OR “reading” OR “reading distance” OR “working distance”.) There were no language restrictions. After deleting duplicate articles, two authors independently screened the studies for inclusion, retrieved potentially relevant studies, and determined study eligibility. Then we reviewed the bibliographies of all selected articles to identify additional studies. Disagreements were resolved by consensus.

### Inclusion and Exclusion Criteria

Near work was defined as the sum of activities with short working distance such as reading, studying, writing, doing homework, watching TV, or playing video games, etc. We included studies that reported any near work activities as covariates with myopia, myopia incidence or progression as the outcome measure. We excluded studies that enrolled subjects older than 18 years of age.

### Data Extraction

For each study, the following characteristics were extracted: (i) last name of first author, (ii) year of publication, (iii) study design, (iv) race/ethnicity of the study population if available, (v) number of subjects in the analysis, (vi) age range of subjects included in the studies, (vii) definition of myopia or myopia progression, (viii) definition of near work. (ix) effect estimate(s), and (x) which confounding factors was adjusted for.

To quantify the effect of near work activity (reading, writing, computer use, and playing video games) on myopia development, some authors computed a weighted variable by adding three times reading, two times computer use, and two times video games use in hours per day as diopter hours [11]. Each article was rated according to the “strength of evidence” as defined by the American Academy of Ophthalmology’s glaucoma panel [17]. Level I indicates that the data provided strong evidence in support of the recommendation, that the design of the study addressed the issue in question, and that the study was performed in the population of interest and executed in a manner that ensured the production of accurate and reliable data, using appropriate statistical methods. Level II indicates that the data provided substantial evidence in support of the recommendation but that the evidence was lacking in some qualities. Level III indicates a consensus of expert opinion in the absence of evidence that met the requirements of levels I and II. Recommendations for clinical outcomes were evaluated as levels A, B, or C [17]. Level A indicates that the recommendation is very important to the clinical outcome; level B indicates that the recommendation is moderately important; and level C indicates that the recommendation is relevant but not critical.

### Statistical Analysis

To evaluate the effect of near work on myopia, the evaluated outcomes included prevalent myopia, myopic development or progression. We reported dichotomous outcomes as odds ratios (ORs) and continuous outcomes as the mean and their respective 95% confidence intervals (CIs). For studies that did not report estimate for general near work, we choose the activity with the shortest working distance to estimate the association. Similarly, we tried to include unadjusted data in our meta-analysis unless only adjusted outcomes being reported. The I^2^ statistic was calculated to determine the proportion of inter-study variation due to heterogeneity, which suggested thresholds for low (25–49%), moderate (50–75%) and high (>75%) values. Study-level data were pooled using a random-effects model [18, 19]. In analyses that included less than 5 studies, a fixed-effects model was used. For any single study with multiple publications (e.g., Singapore Cohort Study of the Risk Factors for Myopia [20–22] and Sydney Myopia Study [20, 23–25]), we only included each set of study participants in the analysis once. We assessed for possible publication bias or a systematic difference between smaller and larger studies with a funnel plot. All statistical analyses were performed with STATA/SE 12.0 (StataCorp LP, College Station, TX). A 2-sided p value less than 0.05 was regarded as significant for all analyses.

## Results

**Fig 1:**
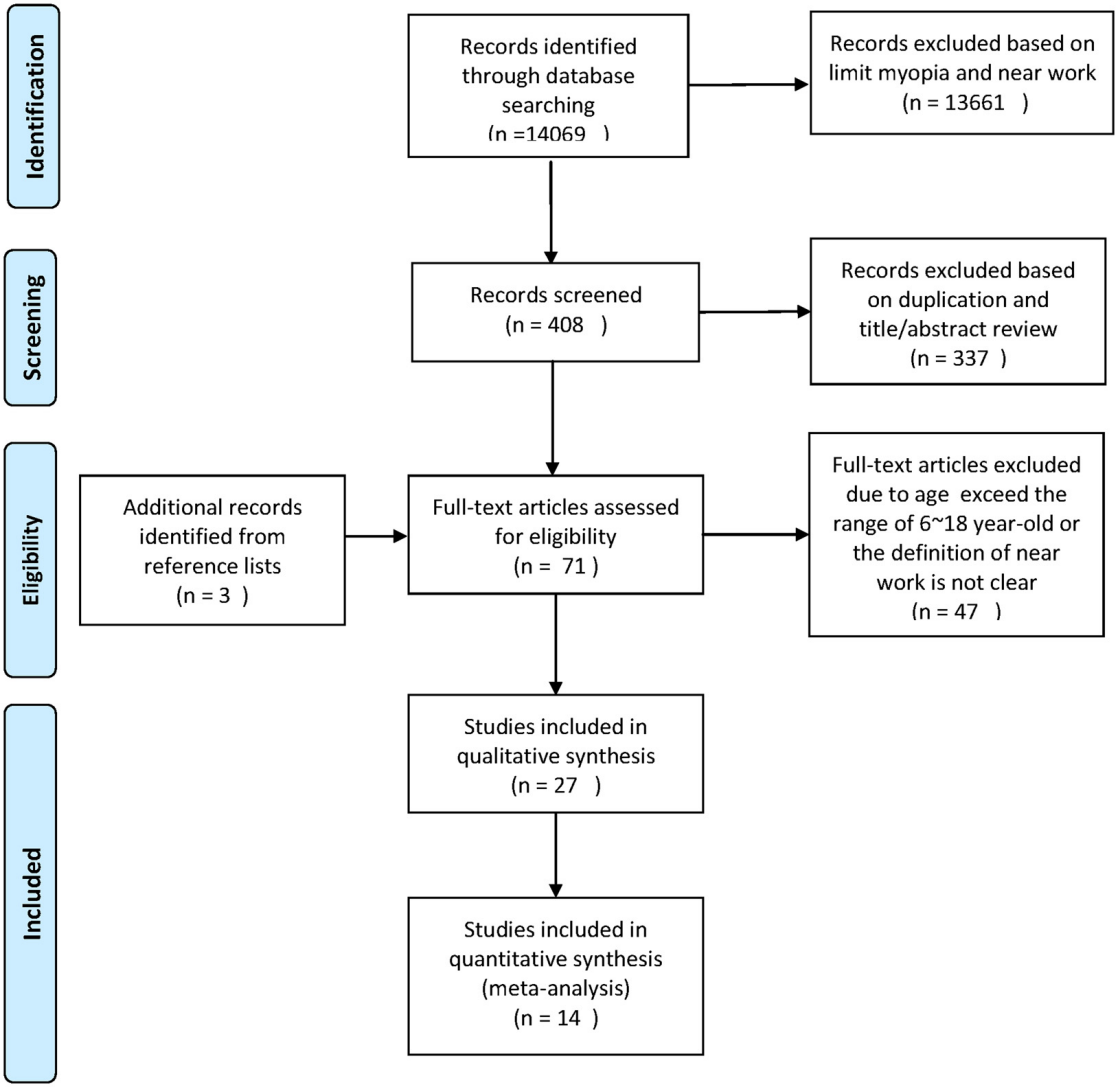
Flow diagram outlining the selection process for the inclusion of studies in the systematic review and meta-analysis. The electronic database searches identified 14,069 citations. After evaluating these citations and their bibliographies, 11 cross-sectional and 3 cohort studies met the inclusion criteria for quantitative analysis for this systemic review. *From*: Moher D, Liberati A, Tetzlaff J, Altman DG, The PRISAM Group (2009). *P*referred *R*eporting *l*tems for Systematic Reviews and *M*eta-*A*nalyses: The PRISAM Statement. PloS Med 6(6): e1000097. doi:10.1371/journal.pmed1000097. For more information, visit www.prisma-statement.org.

The electronic database searches identified 14,069 citations. After evaluating these citations and their bibliographies, 27 studies met the inclusion criteria for this review (Fig 1) [12–14, 20–43]. The importance of clinical outcomes and the characteristics of included studies are summarized in S1–S4 Tables. We included 15 cross-sectional [13, 14, 20, 21, 23, 24, 27–29, 34–36, 41–43] and 12 longitudinal studies [12, 22, 25, 26, 30–33, 37–40]. Fourteen studies were conducted in Asia [12, 14, 20–22, 28, 30, 33–35, 39, 41–43], 6 studies were conducted in North America [13, 29, 31, 32, 38, 40], 3 studies were conducted in Australia [23–25], 3 studies were conducted in Europe [26, 27, 37], and 1 study was conducted in the Middle East [36]. All studies were published between 1989 and 2014 and included a total study population of 25,025 individuals. Time spent on near work was assessed by questionnaires completed by parents, children or both. The definition of myopia varied across studies.

### Estimate of the association between near work activities and prevalence of myopia in cross-sectional studies

Fifteen cross-sectional studies investigated the relationship between near work activities and prevalence of myopia (S2 Table). Ten of them showed that more near work activities appeared to increase the prevalence of myopia in children 6–18 years old [13, 21, 23, 27, 29, 34–36, 41, 42]. Among 210 Chinese children 8–9 years old, Saw et al reported that myopic children performed more total near work activities (2.7± 0.7 hrs/day) than non-myopes (2.3± 1 hrs/day) (*p* = 0.0027) [34]. Among 1,005 7-9-year-old children from the Singapore Cohort Study of the Risk Factors for Myopia (SCORM), those who read more than two books per week had a higher risk (OR = 3.05; 95% CI = 1.80–5.18) of having myopia (spherical equivalent error (SER) of at least ≦- 3.0 diopters [D]) than those who read fewer than two books [21];the result of an analysis of 7-9-year-old Chinese children from both China and Singapore showed that the OR of myopia (SER≦- 0.5) in children who read more than two books per week was 1.43 (95% CI = 1.05–1.94) after adjusting for age, night-light use, parental myopia and country [41]. In 2353 children aged 12–13 years old from the Sydney Myopia Study (SMS), close reading distance (<30 cm) and continuous reading (>30 min) increased the risk of myopia by 2.5-fold (95% CI = 1.74–4.0) and 1.5-fold (95% CI = 1.05–2.10), respectively [23]. Mutti et al found that the multivariate OR of myopia for each diopter-hr per week of near work was 1.02 (95% CI = 1.008–1.032) in 366 American children (mean age of 13.7 years) [13]. In 1378 children aged 15–18 years old from Greece, 43.1% of the myopic children studied >5 hours per day compared with 28.6% of the non-myopic children (χ^2^ = 37.36, p< 0.001) [27]. Deng et al studied 147 American, predominantly white children aged 6–18 years old and found the myopic children watched more television (12.78± 9.28 hrs/week) than non-myopes (8.91± 5.95 hrs/week) (*p* = 0.02) during the school year. In that study, the multivariate OR for the association between myopia and TV viewing was significantly higher than 1.0 [29]. In 377 children aged 6–12 years old from Thailand, the multivariate OR of myopia for each diopter-hr per week of near activities was 1.019 (95% CI = 1.005–1.033) [35]. Khader et al also reported that the odds of having myopia increased by 24% and 16% for each additional 1 hour spent on writing/reading and computer work outside of school, respectively, based on a binary logistic regression analysis in 1777 children aged 12–17 years old from Amman [36]. Among 681 5-13-year-old Chinese children, Guo et al also demonstrated 38% higher odds of having myopia in those who spent more time indoor studying after adjusting for age and maternal myopia [42].

However, 5 other cross-sectional studies did not produce consistent results regarding whether near work activities were risk factors for myopia [14, 20, 24, 28, 43]. Lu et al studied 1,892 children in rural China (mean age of 14.6 years) and found that compared to non-myopic children, myopic children (SER≦-0.5D) did not spend more time on near work activities, including homework (35.3±25.9 versus 34± 24.4 diopter-hrs/week, *p* = 0.62), personal reading (23.8± 24.7 versus 20.7± 21.2 diopter-hrs/week, *p* = 0.12), watching TV (6.8± 5.3 versus 6.2± 5.2 diopter-hrs/week, *p* = 0.22) and playing video games/computer use (18.9± 24.9 versus 21.8± 24.7 diopter-hrs/week, *p* = 0.11) [14]. After adjusting for age, sex, and parental education, none of the near work activities were associated with myopia [14]. Based on a survey by Rose et al involving 4118 children aged 6 and 12 years, the prevalence of myopia was similar among children who spent a low (0–2 hrs/day), moderate (1.6–3.1 hrs/day) or high (>2.6–3.0 hrs/day) amount of time per day on near work activities in both 6- (*p* = 0.08) and 12-year-old (*p* = 0.8) subjects [24]. Rose et al also compared the prevalence of myopia among 6-7-year-old Chinese children who live in Sydney to those who live in Singapore and found only 3.3% myopic (SER≦-0.5D) Chinese children living in Sydney compared to 29.1% living in Singapore. Interestingly, children who live in Sydney even spent more time on total near-work activities (29.93± 20.09 hrs/week) than those who live in Singapore (23.54± 11.84 hrs/week) [20]. Among 145 Taiwanese children who live in rural areas, Wu et al found a weak correlation between myopia and television watching (*p* = 0.059) and no significant associations with other near work activities (reading/writing, computer, and playing piano, etc) [28]. Lin et al studied 386 children living in inner city Beijing and found that children aged 6–12 years with a high level of near work time (3.86–8 hrs/day) did not exhibit significantly more myopic refraction than children with moderate (2.79–3.85 hrs/day) and low (<2.79 hrs/day) levels of near work time after adjusting for the children’s age, gender, average parental refractive error, and time spent on outdoor activities (p trend = 0.94), as well as in children aged 13–17 years (p trend = 0.63) [43].

### Estimate of the association between near work activities and incidence of myopia in cohort studies

Six cohort studies investigated the relationship between near work activities and the incidence of myopia (S3 Table). Only two reported that more near work activities increased the risk of developing myopia [25, 31], but others found no correlation between near work activities and the incidence of myopia [22, 32, 37, 39]. In 1329 children aged 6–14 years old from the Collaborative Longitudinal Evaluation of Ethnicity and Refractive Error (CLEERE), the mean difference in near work was 3.0 more diopter-hrs per week in the became-myopic group (<-0.75D) 1 year before the onset of myopia, which is an increase of 1.7 diopter-hrs per week over the prior year [31]. French et al also reported 5~6 years of follow-up in 2103 children aged 6 and 12 years old at baseline from an SMS database and demonstrated that children who became myopic (≦-0.5D) performed significantly more near work (19.4 vs. 17.6 hrs/week; *p* = 0.02) in the younger cohort (6 y/o at baseline) [25]. In contrast, Saw et al studied 994 Chinese children aged 7–9 years old from Singapore and found that the multivariate risk ratio (RR) of incident myopia was 0.99 (95% CI: 0.97–1.01) per diopter-hr per week of near work [22]. In 514 children aged 8–9 years old from the Orinda Longitudinal Study of Myopia (OLSM), Jones et al recorded the refractive status for 5 years and inquired about the time spent on various activities in the past. They found that children in the myopic shift group did not spend more time (39.49± 20.79 diopter-hrs/week) at near-work activity than the non-myopic group (39.22± 19.67 diopter-hrs/week)(*p* = 0.90) [32]. Among children older than 11 years from the Avon Longitudinal Study of Parents and Children (ALSPAC), those who spent greater than 3 hours per day reading had a relatively higher risk of developing myopia compared to those who spent less than 3 hours per day reading (hazard ratio, HR = 1.22, 95% CI = 0.96–1.55, *p* = 0.098) [37]. Wu et al also studied 571 children aged 7–11 years old from the suburban area of southern Taiwan and reported that near-work activities such as reading, writing, computer use, piano playing, painting or television watching did not increase the risk of developing myopia in non-myopic school children after adjusting for ROC (recess outside the classroom) program and school years [39].

### Estimate of the association between near work activities and myopia progression in prospective studies

Six longitudinal studies demonstrated the relationship between near work activities and the progression of myopia (S4 Table). Two longitudinal studies suggested that near-work activities were risk factors for myopic progression [26, 30], but the findings of other studies did not support that conclusion [12, 33, 38, 40]. Among 238 children with a mean age of 10.9 years, Pärssinen et al found that the faster progression group (-2.9± 0.6D) had a significantly shorter reading distance (22± 3.8 cm) and longer time spent on reading and near work activities (3.5± 0.9 hrs/day) than the slower progression group (-0.5± 0.3D) (24.1± 4.3 cm, 2.9± 0.8 hrs/day, respectively) over a 3-year follow-up [26]. Similarly, Hepsen et al studied 117 Turkish boys with a mean age of 12.9 years old and found that 48.8% of the children with a mean of 6 hrs/day of reading and near work activities had a myopic shift over 3 years compared with only 18.9% boys who displayed a myopic shift in the control group [30]. However, among 80 Chinese children aged 7–11 years old, Yi et al reported that even though the intervention group (near and middle vision activity <30 hrs/week and outdoor activity >14–15 hrs/ week) had less myopic progression (0.38± 0.15D/ year) than the control group (0.52± 0.19D/year), there was no significant difference between both groups in the time spent on near work activities at the two year follow-up [33]. Saw et al studied 153 6-12-year-old children from Singapore over a period of 2 years and found no statistically significant associations between cycloplegic subjective refraction changes and total or raw near work activities (time spent reading and writing as well as reading distance) after adjusting for age, gender, and parental history of myopia [12]. In 835 children aged 6–14 years old from the CLEERE study, Jones-Jordan et al found that the number of hours at each near work activity, such as reading for pleasure, studying, computer or TV, per week was not significantly associated with annual myopia progression at an a priori level of *p*≦0.01 [38]. Scheiman et al also reported on 469 6–11 children aged 6–11 years from COMET (Correction of Myopia Evaluation Trial) and demonstrated that for each additional hour spent on near work activities per week at baseline, the odds of having stable myopia by age 15 decreased by 2% (*p* = 0.07) [40].

### Evidence Synthesis

**Fig 2:**
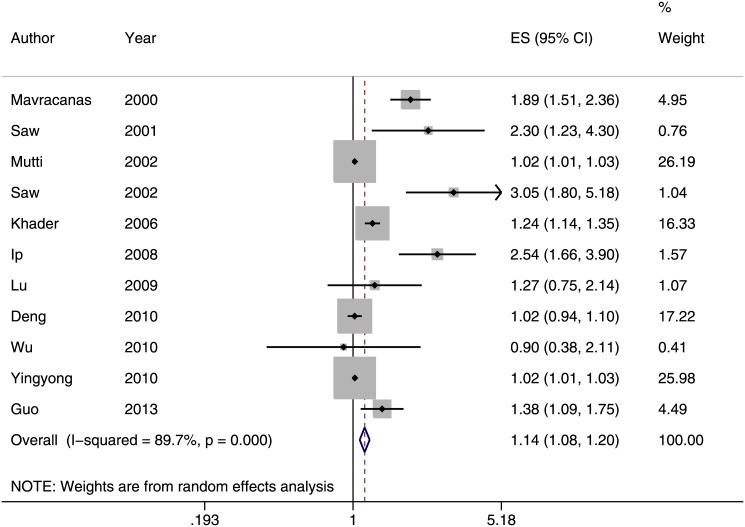
Forest plot of studies reporting an association between near work and prevalence of myopia (odds ratio). A pooled odds ratio of 1.14 (95% CI: 1.08–1.20) suggested that near work was associated with myopia.

**Fig 3:**
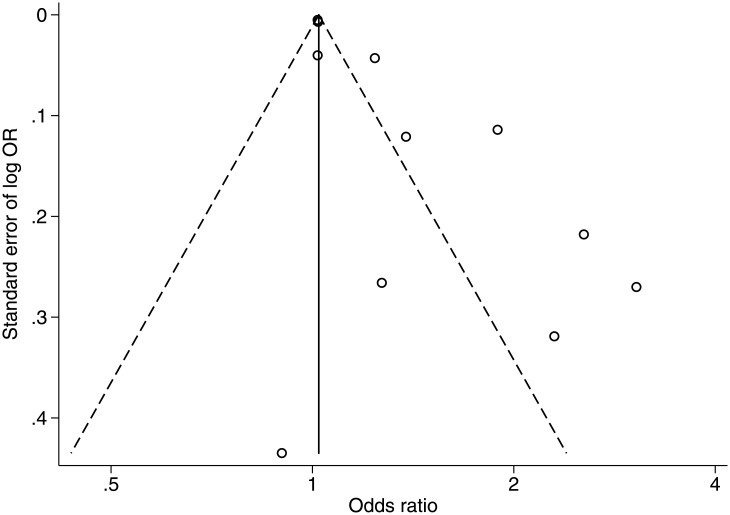
Funnel plot with pseudo 95% confidence limits of studies reporting an association between near work and prevalence of myopia. The funnel plot showed that the distribution of studies was asymmetrical compared to summary estimates.

**Fig 4:**
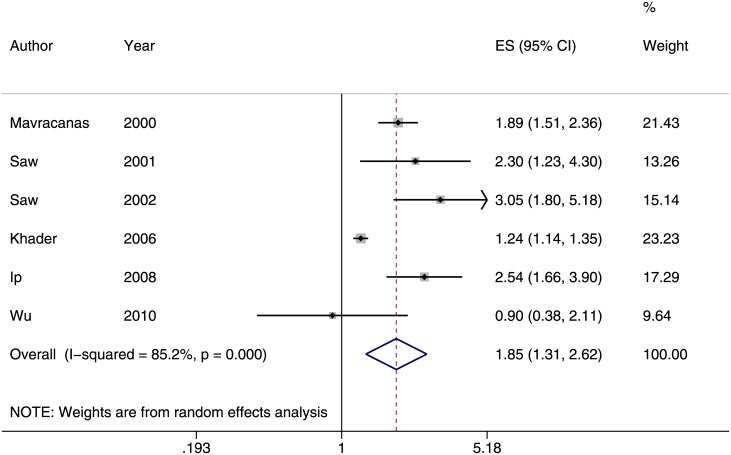
Subgroup of studies reporting the association between near work (dichotomized) and prevalence of myopia (odds ratio). A pooled odds ratio of 1.85 (95% CI: 1.31–2.62) indicated that children who performed more near work were more likely to be myopic.

**Fig 5:**
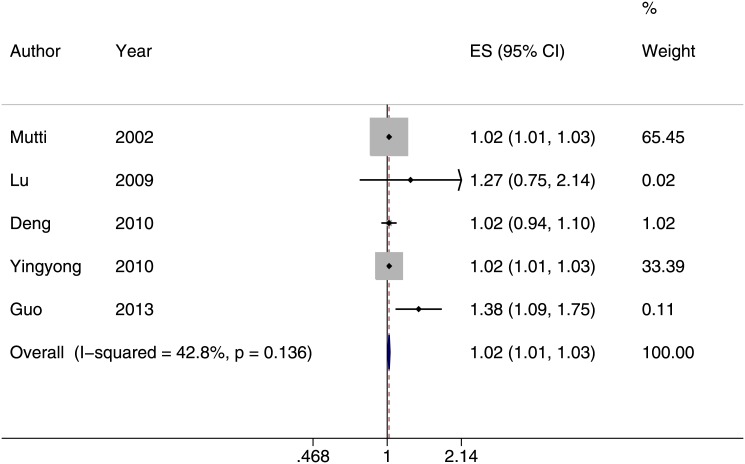
Subgroup of studies reporting an association between near work (per diopter-hour/week) and prevalence of myopia (odds ratio). In children, the odds of myopia increased by 2% (OR:1.02; 95% CI = 1.01–1.03; *I* ^*2*^ = 42.8%) for every one diopter-hour more of near work per week.

**Fig 6:**
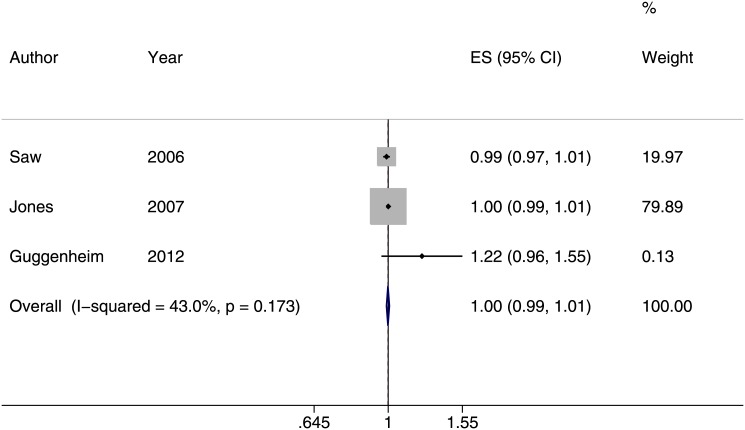
Forest plot of studies reporting an association between near work and the incidence of developing myopia (risk ratio, RR). The incidence of myopia was not increased as children spent more diopter-hours performing near work activities (RR:1.00, 95% CI: 0.99–1.01).

Among the 15 cross-sectional studies, we excluded 4 that did not report odds ratios or their study participants were already included in other studies [20, 24, 41, 43]. This left 11 studies with a total of 10,384 participants for inclusion in the meta-analysis (Fig 2) [13, 14, 21, 23, 27–29, 34–36, 42]. The forest plot showed a pooled odds ratio of 1.14 (95% CI: 1.08–1.20), suggesting that near work is associated with myopia. However, near work was defined differently across studies, and this might explain some of the heterogeneity (*I* ^*2*^ = 89.7%). The funnel plot showed that the distribution of studies was asymmetrical compared to the summary estimate (*p* = 0.003, Fig 3). This result might be explained by potential publication bias or small study effect. We also conducted two separate subgroup analyses based on the definition of near work. Children who performed more near work were more likely to be myopic, (Odds ratio [OR] = 1.85; 95% confidence interval [CI] = 1.31–2.62; *I* ^*2*^ = 85%, Fig 4) [21, 23, 27, 28, 34, 36] and the odds of myopia increased by 2% (OR:1.02; 95% CI = 1.01–1.03; *I* ^*2*^ = 42.8%) for every one diopter-hour more of near work per week (Fig 5) [13, 14, 29, 35, 42]. Three of the six cohort studies reported similar outcome measures in assessing the development of myopia [22, 32, 37]. The meta-analysis estimates showed that the incidence of myopia did not increase with increasing diopter-hrs spent on near work activities (risk ratio of 1.00, 95% CI: 0.99–1.01, *I* ^*2*^ = 43%, Fig 6). Because studies reported outcomes regarding myopic progression very differently, we were not able to combine their results [12, 26, 30, 33, 38, 40]. We also summarized the differences in time spent on near work activities between myopes and non-myopes from four studies [13, 14, 29, 32]. On average, children with myopia spent 0.66 more hours per week on reading (95% CI: 0.16–1.17) compared to those without myopia. Time spent on watching TV, playing computer or video games, and studying was not significantly associated with myopia (Table 1).

**Table 1:** The difference in time spent on near work activities between myopes and non-myopes.

	Mean differences hours per week (95% CI)	Heterogeneity (*I* ^*2*^)
**Reading**	0.66 (0.16 to 1.17)	8%
**Watching TV**	-0.22 (-0.96 to 0.51)	66%
**Playing computer or video games**	0 (-0.60 to 0.57)	54%
**Studying**	-0.01 (-0.60 to 0.57)	71%

## Discussion

The evidence for clinical recommendations is based on a comprehensive search of relevant articles and a consolidation of the available evidence. Because only 1 of the evaluated studies was a randomized controlled trial and all others were observational studies, the “strength of the evidence” for all recommendations was considered to be II. Therefore, recommendations regarding decreased near work activities were considered level B, which is moderately important to outcome (Table 2).

**Table 2:** Clinical recommendations for the different behaviors.

Behavior	Recommendation	Evidence rating
Near work	Decrease the time spent reading to reduce the risk of children developing myopia	II, B

B = moderately important recommendation; II = substantial evidence supporting recommendation but lacking some qualities required for strong support.

Myopia is one of the five immediate priorities for the ‘Vision 2020’ initiative as set out by the World Health Organization (WHO) because it is an important cause of reduced vision in populations throughout the world [44]. The prevalence of myopia around the world has increased recently [45]. Several factors have been suggested to play a role in the development of myopia. Other than genetic factors, environment is also an important contributing factor in the development of myopia. Studies on populations with very similar genetic backgrounds that grew up in different environments have shown that those growing up in rural environments have a lower prevalence of myopia [46]. Environmental influences related to prolonged reading or near work as well as fewer hours spent outdoors are associated with a higher prevalence of myopia. A systematic review and meta-analysis to identify the association between time spent outdoors and myopia indicated a 2% reduced odds of myopia per additional hour of time spent outdoors per week after adjustment for covariates [47]. In our study, the association between near work and myopia indicated a 2% increased odds of myopia per additional diopter-hour of time spent on near work per week. In children lifestyles, outdoor activities and near work might be important antagonistic factors associated with myopia. Further prospective studies are necessary for the balance of near work and outdoor activities in the prevention of myopia.

According to previous studies, juvenile-onset myopia typically develops at six to eight years of age and progresses at a rate of approximately 0.50 D per year through 15 to 16 years of age in Western children and approximately 1 D per year progression in Asian children [48–51]. The progression of myopia is typically faster at younger ages [7, 12, 49, 52, 53]. Therefore, we reviewed articles that studied children aged 6–18 years and evaluated whether near work activities affect myopic incidence, prevalence and progression. In the East, the educational system and stress is different from in the West. Eastern parents pay a lot of attention to the academic performance of children and encourage more time spent on near work. In contrast, Western parents pay more attention to children’s physical education and encourage more outdoor activities. This difference might partly contribute to the high prevalence of myopia in the East [54–56]. Recently, Morgan IG & Rose KA proposed that the extensive use of after-school tutorials and increasing educational load are associated with high prevalence rates of myopia [57]. An association with additional tutorial classes at the primary school level has also been reported in Singapore [58]. In Taiwan, most children are sent to private tutorial classes after school because parents are not available to accompany them and anticipate better academic performance. Additional hours spent in the tutorial classroom most likely increases near work time spent on homework and associated paper work. If a child has 4 hours per day for near work (33 cm) in tutorial classes after school during weekdays (Monday to Friday), then he would most likely have 120% of additional odds of myopia.

The theory of hyperopic defocus from a deficient accommodative response in juvenile myopia is considered to be the association between near work in human myopia and the minus lens results from animal studies [13, 59]. The findings from this meta-analysis indicated that individuals who perform more near work activities had an 80% higher risk of having myopia. In addition, myopic children spent more time reading, but not studying, using a computer, or watching TV, than non-myopic children. (Table 1) This result indicates that the effect of near work on myopia seems to come from reading. The evidence for a link between near work and myopic incidence or progression is not observed based on the interpretation of the results from cohort studies and the 1 RCT. In addition to the fact that the number of available prospective studies is considerably low, a number of factors may contribute to the inconsistent conclusions; these factors include differences in study design, children’s ethnicity, definitions of myopia and near work, accuracy of the self-reported or parent-reported activity times, and quality of the data collection. In addition, the impact of near work on myopia may be cumulative over time, and lighting or temporal factors (e.g., break time between texts) may also affect final estimations. Given that these findings are limited to only a few heterogeneous studies, we recommend that more longitudinal and randomized controlled trials should be performed to confirm whether near work is a risk factor for the development of myopia.

In conclusion, this systematic review shows that near work activities were associated with myopia and that increased diopter-hrs of near work might increase myopia prevalence.

## Supporting Information

S1 PRISMA Checklist (DOC)

S1 Table Patient and study characteristics of the total included studies. (DOC)

S2 Table Cross sectional studies investigating the correlation between near work activities and myopia. (DOC)

S3 Table Cohort studies investigating the relationship between near work activities and incidence of myopia. (DOC)

S4 Table Studies investigating the correlation between near work activities and the progression of myopia. (DOC)
