# Supplementary material for: The Association between Near Work Activities and Myopia in Children—A Systematic Review and Meta-Analysis
Source: PLoS One. 2015 Oct 20;10(10):e0140419. doi: 10.1371/journal.pone.0140419 (PMC4618477; doi:10.1371/journal.pone.0140419)
Supplement: S1 Table — (DOC) [file pone.0140419.s002.doc]

|  | **S1 Table. Patient and Study Characteristics of the Total Included Studies** | | | | | | | | |
| --- | --- | --- | --- | --- | --- | --- | --- | --- | --- |
| Source | | Country | Race/Ethnicity | Methods ( for prevalence (Pre)/ incidence (I)/ progression (P) of myopia) | Strength of evidence | Study population | Age | Outcome summery |  |
| Mavracanas et al (2000)[27] | | Northern Greece |  | Cross sectional (Pre) | II | 1431 | 15~18 | **Myopia correlates strongly with near work** |  |
| Saw et al (2001)[34] | | China |  | Cross sectional (Pre) | II | 210 | 8~9 | **The prevalence of myopia correlates with reading and writing** |  |
| Mutti et al (2002)[13] | | USA |  | Cross sectional (Pre) | II | 366 | mean 13.7 | **Near work is associated with myopia** |  |
| Saw et al, SCORM (2002)[21] | | Singapore | Chinese 72.5% | Cross sectional (Pre) | II | 1005 | 7~9 | **Children with a greater current reading exposure were more likely to be myopic** |  |
| Saw et al, (2002)[41] | | Singapore and China | Chinese | Cross sectional (Pre) | II | Singapore: 729  China: 369 | 7~9 | **Reading may be associated with higher myopia** |  |
| Khader et al (2006)[36] | | Amman |  | Cross sectional (Pre) | II | 1777 | 12~17 | **Myopia was significantly associated with computer use, reading and writing** |  |
| Ip et al, SMS (2008)[23] | | Australia | Predominantly European Caucasians (64.5%); East Asian (15.0%). | Cross sectional (Pre) | II | 2353 | 11.1~14.4 | **Near work such**  **as close reading distance (< 30 cm) and continuous reading (> 30 minutes) independently increased the odds of having myopia** |  |
| Rose et al (2008)[24] | | Australia | Majority European Caucasians | Cross sectional (Pre) | II | 6-year-old: 1765 12-year-old: 2353 | mean 6.7 | After adjusting for gender, ethnicity, parental myopia, outdoor activity, and other risk factors, there was no association of near work and myopia |  |
| *Rose et al (2008)[20]* | | *Singapore and Australia* | *Chinese* | *Cross sectional* (Pre) | *II* | *Sydney:128*  *Singpore: 628* | *6 and 7* | *Near work is not associated with prevalence of myopia* |  |
| Lu et al, The Xichang Pediatric Refractive Error Study (2009)[14) | | China |  | Cross sectional (Pre) | II | 1892 | mean 14.6 | Time and diopter- hours spent on near activities did not differ between children with and without myopia. |  |
| Deng et al (2010)[29] | | USA | 95% white | Cross sectional (Pre) | II | 147 | 6~18 | Myopes watched more television, not more reading or studying than non-myopes. |  |
| Penpimol Yingyong (2010)[35] | | Thailand |  | Cross sectional (Pre) | II | 377 | 6-12 | **Increase hours of near work was significantly associated with myopia** |  |
| Wu et al (2010)[28] | | Taiwan |  | Cross sectional (Pre) | II | 145 | 7~12 | Near work is not associated with myopia |  |
| Guo et al (2013)[42] | | China |  | Cross sectional (Pre) | II | 6-year-old: 382  9-year-old: 299 | 5~13 | **Presence of myopia was associated with more time spent indoors studying** |  |
| Lin et al, BMPS (2014)[43] | | China |  | Cross sectional (Pre) | II | 386 | 6-17 | Children with more near work time did not exhibit a significantly more myopic refraction after adjusting for gender, outdoor activity time, and average parental refractive error. |  |
| Saw et al, SCORM (2006)[22] | | Singapore | Chinese | Cohort (I) | II | 994 | 7~9 | Near work was not associated with incident myopia. |  |
| Jones et al, OLSM (2007)[32] | | USA | Majority white | Cohort (I) | II | 514 | 8~9 | Near work was not associated with incident myopia. |  |
| Jones-Jordan et al, CLEERE study(2011)[31] | | USA | Diverse | Cohort (I) | II | 1329 | 6~14 | **Hours per week spent reading or using a computer/playing video games were signiﬁcantly greater in myopes than in emmetropes at onset,** not before |  |
| Guggenheim et al, ALSPAC (2012)[37] | | England | White | Cohort (I) | II | 9109 | 7~15 | The association between reading and incident myopia was controversial. |  |

| French et al, SMS (2013)[25] | Australian |  | Cohort (I) | II | 6-year-old: 892  12-year-old: 1211 | 6~18 | **Children who became myopic performed signiﬁcantly more near work in the younger cohort** |
| --- | --- | --- | --- | --- | --- | --- | --- |
| Wu et al (2013)[39] | Taiwan |  | Cohort (I) | II | 571 | 7~11 | Reading and writing activity, computer use, other near-work activities, and television were not statistically signiﬁcant associated with myopia shift in nonmyopic schoolchildren |
| Parssinen et.al (1993)[26] | Finland |  | Cohort (P) | II | 238 | mean 10.9 | **Myopic progression and final myopia were related to time spent on reading and close work and to reading distance** |
| Saw et al (2000)[12] | Singapore |  | Cohort (P) | II | 153 | 6~12 | Near-work activity was not related  to myopia progression. |
| Hepsen et al (2001)[30] | Turkey |  | Cohort (P) | II | 117, boys | mean 12.9 | **Reading and near work might cause refractive myopic shifts in emmetropic students.** |
| Ｙi et al (2011)[33] | China |  | RCT (P) | I | 80 | 7~11 | Near-work activity was not related  to myopia progression. |

| Jones-Jordan et al, CLEERE study (2012)[38] | USA | Diverse | Cohort (P) | II | 835 | 6~14 | Near work had little meaningful effect on the rate of myopia progression. |
| --- | --- | --- | --- | --- | --- | --- | --- |
| Scheiman et al, COMET (2014)[40] | USA |  | Cohort (P) | II | 479 | 6~11 | less near work activity might potentially, not significantly be associated with myopia stabilisation by age 15. |

*Italic type: not included in statitcs;* Bald: statistical significant correlation

SCORM: Singapore Cohort Study of the Risk Factors for Myopia; SMS: Sydney Myopia Study; CLEERE: Collaborative Longitudinal Evaluation of Ethnicity and Refractive Error; OLSM: Orinda Longitudinal Study of Myopia; ALSPAC: Avon Longitudinal Study of Parents and Children; BMPS: Beijing Myopia Progression Study RCT: randomized controlled trial; COMET: Correction of Myopia Evaluation Trial
