# Supplementary material for: The Association between Near Work Activities and Myopia in Children—A Systematic Review and Meta-Analysis
Source: PLoS One. 2015 Oct 20;10(10):e0140419. doi: 10.1371/journal.pone.0140419 (PMC4618477; doi:10.1371/journal.pone.0140419)
Supplement: S2 Table — (DOC) [file pone.0140419.s003.doc]

| **S2 Table. Cross sectional studies investigating the correlation between near work activities and myopia** | | | | | | | | | |
| --- | --- | --- | --- | --- | --- | --- | --- | --- | --- |
| Source | Cycloplegia | Information | Myopia definition | Near work activity definition | Odds Ratio(OR) (95%CI) (p<0.05) | Adjusted Covariates | Mean+/-SD(p<0.05) |  | |
| Mavracanas et al (2000)[27] | Not found | questionnaire | Myopia: SER≦- 0.25D | Studying (hrs/day) |  |  | Myopia: Non-myopia= 4.3 : 3.6 |  | |
|  |  |  |  | Studying >5hrs/day |  |  | The population percentage of myopia: Non-myopia: 43.14%: 28.62% |  | |
| Mutti et al (2002)[13] | Y |  | Myopia: SER≦-0.75D | Study(hrs/week) |  |  | Myopia: Emmetropia= 11.2± 7.2: 8.9± 5.2 |  | |
|  |  |  |  | *Video/computer(hrs/week)* |  |  | *Myopia: Emmetropia= 2.7± 4.1: 2.2 ± 3.2* |  | |
|  |  |  |  | Reading for pleasure (hrs/week) |  |  | Myopia: Emmetropia= 5.8 ± 4.8: 4.1± 4.6 |  | |
|  |  |  |  | *TV(hrs/week)* |  |  | *Myopia: Emmetropia= 9.2± 6.8: 8.3± 5.7* |  | |
|  |  |  |  | Near work (dioper-hrs/week) | 1.018 (1.008–1.027) | parents myopia, scores, outdoor activity | Myopia: Emmetropia= 65.1± 34.1: 51.5± 24.4 |  | |
| Saw et al (2001)[34] | Y | questionnaire | Myopia: SER＜-0.50D | Total reading and writing in Chinese and English (hrs/day)  *Computer use (hrs/day)*  Total near-work activity(hrs/day) |  |  | Myopia: No Myopia= 2.3± 0.6: 1.9± 0.7  *Myopia: No Myopia= 0.04± 0.2: 0.04± 0.2*  Myopia: No Myopia= 2.7± 0.7: 2.3± 1 |  | |
| Saw et al, SCORM (2002)[21] | Y | Parents completed questionnaire | Myopia: SER<-0.5 D  higher myopes (SER≦-3.0D) lower myopes ( -3.0< SER≦0.5 D),  nonmyopes (SER > -0.5D). | Reading more than two books/week | O.R for higher myopia (>-3.0D): 3.05 (1.80–5.18) | age, gender, race, night light, parental myopia, and school | Higher:Lower: Nonmyopia in bookes read/week= 4.3±5.8: 2.6±2.5: 2.5±2.2 |  | |
|  |  |  |  | Read more than 2 hrs/ day | O.R for higher myopia: 1.50 (0.87–2.55) | age, gender, race, night light, parental myopia, and school | Reported reading hours/ day= 2.8±1.1: 2.3±1.1: 2.4±1.3 |  | |
| | Saw et al (2002)[41] | Y | Parents completed questionnaire | Myopia: SER<-0.5 D  higher myopes (SER≦-3.0D) lower myopes ( -3.0< SER≦0.5 D),  nonmyopes (SER > -0.5D). | Reading more than two books/week | O.R for myopia (<-0.5D): 1.43 (1.05–1.94)  O.R for higher myopia (≦-3.0D): 2.81 (1.69–4.69) | age, night-light use, parental myopia, country | Higher:Lower: Nonmyopia in books read/week=  3.6±2.6: 2.6±2.3: 2.1±2.0 | | --- | --- | --- | --- | --- | --- | --- | --- | | Khader et al (2006)[36] | Not found | Parents and children completed questionnaire | Myopia: SER≦  - 0.50 D | Reading and writing at home | O.R for myopia: 1.24 (1.14–1.35) | age, gender, ethnicity, and school type. |  | |  |  |  |  | Computer using | OR for myopia: 1.16 (1.06–1.26) | age, gender, ethnicity, and school type. |  | |  |  |  |  | *Watching TV*  *(hrs/day)* |  |  | *Myopia: No Myopia=*  *1.71± 0.48: 1.8± 0.79* | | | | | | | | |  | |
| Ip et al, SMS (2008)[23] | Y | children completed questionnaire | Myopia: SER<  - 0.50 D | Continuous reading > 30 minutes | O.R for myopia: 1.5 (1.05–2.1) | age, gender, ethnicity, and school type. |  |  | |
|  |  |  |  | Close reading distance < 30 cm | OR for myopia: 2.5 (1.7–4.0) | age, gender, ethnicity, and school type. |  |  | |
| Rose et al (2008)[24] | Y | Parents complete questionnaire | Myopia: SER≦- 0.50 D | *The mean refractive error in low: moderate: high near work activity in*  ***6 year-old*** *children* |  | gender, ethnicity, parental myopia, parental employment, and education | *+ 1.35: + 1.32: + 1.28* |  | |
|  |  |  |  | ***12 year-old*** *children* |  | gender, ethnicity, parental myopia, parental employment, and education | *+ 0.48: + 0.48: +0.42* |  | |
| Rose et al (2008)[20] | Y | parents completed questionnaires | Myopia: SER≦- 0.50 D | Myopia(%), Sydney: Singapore |  |  | 3.3: 29.1 |  | |
|  |  |  |  | Books read,(No./week), |  |  | 4.44 ± 2.46: 2.39± 2.27 |  | |
|  |  |  |  | Reading and writing (hrs/week). |  |  | 20.81± 13.88: 17.76± 8.78 |  | |
|  |  |  |  | *Computer use, (hrs/week),* |  |  | *4.65± 6.62: 3.55± 4.48,* |  | |
|  |  |  |  | Total near-work activity (hrs/week), |  |  | 29.93± 20.09: 23.54± 11.84 |  | |
|  |  |  |  | *TV viewing ( hrs/week),* |  |  | *11.32± 1.47: 12.65± 7.37,* |  | |
| Lu et al, The Xichang Pediatric Refractive Error Study (2009)[14] | Y | questionnaire | Myopia: SER≦- 0.50 D | *Homework, (diopter-hrs/week)* | *O.R for myopia: 1.11 (0.60-2.05)* |  | *Myopia: Non-myopia= 35.3± 25.9: 34.0± 24.4* |  | |
|  |  |  |  | *Personal reading, (diopter-hrs/week)* | *O.R for myopia: 1.27 (0.75-2.13)* |  | *Myopia: Non-myopia= 23.87± 24.7: 20.7± 21.2* |  | |
|  |  |  |  | *Watching television, (diopter-hrs/week)* | *O.R for myopia: 1.41 (0.82-2.41)* |  | *Myopia: Non-myopia= 6.8± 5.3: 6.2± 5.2* |  | |
| Wu et al (2010)[28] | Y | Parents completed question | Myopia: SER≦- 0.75 D | *Watching TV*  *(often or seldom)* | *3.0 ( 1.0–9.2),* | # |  |  | |
|  |  |  |  | *Reading/writing*  *(often or seldom)* | *0.9 (0.4–2.2)* | # |  |  | |
|  |  |  |  | *Computer*  *(often or seldom)* | *1.0(0.4–2.5),* | # |  |  | |
|  |  |  |  | *Other near work activities (eg, plays piano/violin, calligraphy and painting)(often or seldom)* | *0.7 (0.2–2.6),* | # |  |  | |
| Deng et al (2010)[29] | N | questionnaire | Myopia: SER≦- 0.50 D at the time of the survey | *Study(hrs/week) in school year* | *1.018 (0.940–1.101)* | *survey age and number of myopic parents.* | *Myopia:Non-myopia= 10.8± 5.58: 9.14± 7.29* |  | |
|  |  |  |  | *Video/computer(hrs/week)in school year* | *1.028 (0.946–1.117)* | *survey age and number of myopic parents.* | *Myopia:Non-myopia= 6.00± 5.76: 4.96± 4.58* |  | |
|  |  |  |  | *Reading for pleasure (hrs/week) in school year* | *1.035 (0.929–1.154)* | *survey age and number of myopic parents.* | *Myopia: Non-myopia= 5.60± 4.37: 4.95± 3.63* |  | |
|  |  |  |  | TV(hrs/week) in school year | 1.069 (1.010–1.132) | *survey age and number of myopic parents.* | *Myopia: Non-myopia= 12.78± 9.28: 8.91± 5.95* |  | |
| Penpimol Yingyong (2010)[35] | Y | questionnaire | Myopia: SER≦-0.50D | Studying (hrs/week)  Reading for pleasure(hrs/week)  *Watching TV(hrs/week)*  *Computer(hrs/week)*  Near activities  (dioper-hrs/week) | 1.019 (1.005-1.033) | parents myopia | Myopia: Emmetropia= 11.3± 7.1: 8.7± 5.3  Myopia: Emmetropia= 5.9± 4.7: 4.0± 4.7  *Myopia: Emmetropia= 9.3± 6.7: 8.1± 2.2*  *Myopia: Emmetropia= 2.8± 4.0: 2.0± 3.3*  Myopia: Emmetropia= 66.0± 33.7: 50.1± 23.7 |  | |
| Guo et al (2013)[42] | N | questionnaire | Myopia: SER≦-1.0D | Studying  Viewing television  *Using electric gadgets* | 1.38 (1.09–1.75)  0.62 (0.48–0.81)  *0.84 (0.42–1.69)* | age, maternal myopia |  | |  |
| Lin et al (2014)[43] | Y | questionnaire | Myopia: SER≦-0.50D | *The mean refractive error in low: moderate: high near work activity in* ***6-12 year-old*** *children*  ***13-17 year-old*** *children* |  | *age, gender, average parental refractive error, time spent on outdoor activity* | - *0.59 ± 2.62: - 0.63± 2.21:*   *- 0.70± 1.92*  *- 2.80 ± 2.05: -2.89± 1.95: -3.13± 2.18* | |  |

*Italic type: no statistical significance*

#: adjust for school year, gender, myopic parents, computer, other near work activities, outdoor activity; SCORM: Singapore Cohort Study of the Risk Factors for Myopia; SER: spherical equivalent refractive error
