# Supplementary material for: The Association between Near Work Activities and Myopia in Children—A Systematic Review and Meta-Analysis
Source: PLoS One. 2015 Oct 20;10(10):e0140419. doi: 10.1371/journal.pone.0140419 (PMC4618477; doi:10.1371/journal.pone.0140419)
Supplement: S3 Table — (DOC) [file pone.0140419.s004.doc]

| **S3 Table. Cohort studies investigating the relationship between near work activities and incidence of myopia** | | | | | | | | | | |  |
| --- | --- | --- | --- | --- | --- | --- | --- | --- | --- | --- | --- |
| Source | Cycloplegia | F/U (years) | Information | Myopia definition | Intervention | near work activity definition | Odds Ratio (95%CI) | Adjusted Covariates | Mean+/-SD (p<0.05) | |  |
| Saw et al, SCORM (2006)[22] | Y | 3 | Parents completed questions | Myopia: SER≦- 0.75 D |  | *Books read per week* | *R.R for incident of myopia:*  *1.01 (0.97–1.05)* | Age, gender, parental myopia, parental income | |  | |
|  |  |  |  |  |  | *Reading (hrs/day)* | *0.99 (0.92–1.07)* |  | |  | |
|  |  |  |  |  |  | *Computer use* | *0.94 (0.77–1.15)* |  | |  | |
|  |  |  |  |  |  | *Video game playing (hrs/day)* | *0.94 (0.8–1.09)* |  | |  | |
|  |  |  |  |  |  | *Watching TV (hrs/day)* | *0.96 (0.87–1.06)* |  | |  | |
|  |  |  |  |  |  | *Diopter-hours per week* | *0.99 (0.97–1.01)* |  | |  | |
|  |  |  |  |  |  | *Total near work (hrs/day)* | *0.97 (0.93–1.01)* |  | |  | |
| Jones et al, OLSM (2007)[32] | Y | 5 | Parents completed questions | Myopia: SER≦- 0.75 D |  | *Reading (hrs/week)* | *O.R for future myopia: 1.04 (0.99–1.10)* |  | | *Future myopia: Non-myopia= 4.61± 3.66: 4.06± 3.48* | |
|  |  |  |  |  |  | *TV(hrs/week)* | *O.R for future myopia: 0.97 ( 0.93–1.01)* |  | | *Future myopia: Non-myopia= 7.63± 5.17: 8.46± 5.66* | |
|  |  |  |  |  |  | *Studying (hrs/week)* | *O.R for future myopia: 0.98 (0.93–1.04)* |  | | *Future myopia: Non-myopia= 4.32± 4.00: 4.56± 3.97* | |
|  |  |  |  |  |  | *Computer/video games (hrs/week)* | *O.R for future myopia: 1.01 (0.94–1.09)* |  | | *Future myopia: Non-myopia= 2.52± 2.92: 2.45± 2.81* | |
|  |  |  |  |  |  | *Near-work (diopter-hrs/ week)* | *O.R for future myopia: 1.00 (0.99–1.01)* |  | | *Future myopia: Non-myopia= 39.49± 20.79: 39.22± 19.67* | |
| Jones-Jordan et al, CLEERE study(2011) [31] | Y | 10 | Parents complete questionnaire | Myopia: SER≦  - 0.75 D Emmetropia: SER -0.25~ +1.0 D |  | Reading (hrs/week) | Mean difference when myopia onset:  0.70 (0.31-1.18) |  | |  | |
|  |  |  |  |  |  | Studying (hrs/week) | 0.52 (0.04 - 1.00) |  | |  | |
|  |  |  |  |  |  | Computer/Vedio games(hrs/week) | 0.81 (0.40 - 1.22) |  | |  | |
|  |  |  |  |  |  | Watching TV (hrs/week) | 1.31 (0.51- 2.10) |  | |  | |
|  |  |  |  |  |  | Near work (diopter-hrs /week) | 6.10 (3.39- 8.81) |  | |  | |
| Guggenheim et al, ALSPAC (2012)[37] | N | 8 | Parents completed questhionnaires | Myopic ≤ -1 D Emmetropic/  hyperopic ≥–0.25 D |  | *Reading: high (>3 hrs/day), vs low(<3 hrs/day) amount of time* | *H.R (95% CI) for incident myopia after age 11: 1.22 (0.96–1.55)* | *H.R adjust with the number of myopic parents, time spent outdoors, and gender* | |  | |
| French et al, SMS (2013)[25] | Y | 5~6 | questhionnaires | Myopia: SER≦  - 0.5 D |  | Near work (diopter-hrs /week) |  |  | | Incident myopia: Non-myopia= 49.73: 43.21 in cohort with aged 6 years at baseline | |
| Wu et al (2013)[39] | Y | 1 | Parents completed questhionnaires | Myopia: SER≦  - 0.5 D |  | *Reading/writing (frequent vs.*  *seldom or none)*  *Computer (frequent vs.*  *seldom or none)*  *Other near work (playing piano or painting…; frequent*  *vs. seldom or none)*  *TV (frequent vs. seldom or*  *none)* | *Myopic Shift*  *(D/yr) in nonmyopic children*  *-0.04 ( 0.20 to 0.11)*  *0.10 (- 0.08 to*  *0.27)*  *- 0.03 (-0.21 to 0.15)*  *-0.03 (-0.32 to 0.24)* | gender, myopic parents, baseline  SER, reading and writing activity, computer use, other near-work  activities, television, and outdoor activity after school, ROC program | |  | |

*Italic type: no statistical significance*

SCORM: Singapore Cohort Study of the Risk Factors for Myopia; OLSM: Orinda Longitudinal Study of Myopia; CLEERE: Collaborative Longitudinal Evaluation of Ethnicity and Refractive Error; SER: spherical equivalent refractive error; ROC: recess outside the classroom
