# Supplementary material for: The Association between Near Work Activities and Myopia in Children—A Systematic Review and Meta-Analysis
Source: PLoS One. 2015 Oct 20;10(10):e0140419. doi: 10.1371/journal.pone.0140419 (PMC4618477; doi:10.1371/journal.pone.0140419)
Supplement: S4 Table — (DOC) [file pone.0140419.s005.doc]

| **S4 Table. Studies investigating the correlation between near work activities and progression of myopia** | | | | | | | | | | |
| --- | --- | --- | --- | --- | --- | --- | --- | --- | --- | --- |
| Source | Cycloplegia | F/U (years) | Information | Myopia definition | Intervention | near work activity definition | Diopter (95%CI) | Adjusted Covariates | Mean+/-SD (p<0.05) |  |
| | Saw et al  (2000)[12] | Y | 2 | Parents completed questionnaire | Myopia: sphere -1.0 to -4.0 D  Myopia progression:  difference between final and initial refractive error measurement in diopters /year |  | *Raw total near work ± hrs per day on school weekday* | *Regression coefficient (95%CI)=*  *0.023 (-0.18 to 0.063)* | adjust for age, gender, and parental myopia (Rate of change in cycloplegic subjective refraction readings as the response variable and the factors as the main exposures of interest |  | | --- | --- | --- | --- | --- | --- | --- | --- | --- | --- | |  |  |  |  |  |  | *Raw total reading and writing ± hrs per day on school weekly* | *Regression coefficient (95%CI)=*  *0.028 (-0.027 to 0.083)* |  | |  |  |  |  |  |  | *Distance of eye from book while reading or writing* | *Regression coefficient (95%CI)=*  *0.0087(-0.004 to 0.022)* |  |  | Hepsen et al (2001)[30] | Y | 3 | Questionnaire | Myopic progression: >-0.5D | In group 1, the average daily mean time spent on reading and close-work was 6 hrs |  |  |  | The prevalence rate of myopic progression in 3 years: group 1: group2= 48.8% : 18.9% | | --- | --- | --- | --- | --- | --- | --- | --- | --- | --- | | | | | | | | | | |  |
| Parssinen et.al (1993 )[26] | Y | 3 | Parents completed questionnaire | Slow progression: -0.5± 0.3D Fast progression: -2.9±0.6D |  | Time spent on reading (hrs/day) |  |  | slow vs fast progression: 1.2 vs1.4 |  |
|  |  |  |  |  |  | Time spent on reading and close work (hrs/day) |  |  | slow vs fast progression : 2.9±0.8 vs 3.5±0.9 |  |
|  |  |  |  |  |  | Time spent outdoors (hrs/day) |  |  | slow vs fast progression : 3.2±1.4vs2.5±1.1 |  |
|  |  |  |  |  |  | Reading distance (cm) |  |  | slow vs fast progression : 24.1±4.3vs22.0±3.8 |  |
| Ｙi et al (2011)[33] | Y | 2 | Parents and children together completed questionnaire | Interventrion group: 0.38± 0.15D/ year  Control group: 0.52± 0.19 D/ year | Near and middle vision activiry <30 hrs/week and outddor activity >14-15hrs/ week | Watching TV(hrs/week) |  |  | Control vs Intervenion: 11.8±1.9 vs 7.3±1.8  *(No significance after multi-logistic regression)* |  |
| Jones-Jordan et al, CLEERE study(2012) [38] | Y | 1 | Parents completed questionnaire | Myopia: SER≦  - 0.75 D | average of 10 additional hours of activity per  week on myopia progression in 1 year | *Reading for pleasure*  *Studying*  *Computer/video games*  *TV*  *Diopter-hours* | *-0.07 (-0.14 to 0.003)*  *0.004 (-0.06 to 0.07)*  *-0.05 (-0.13 to 0.04)*  *-0.006 (-0.05 to 0.04)*  *-0.007 (-0.02 to 0.004)* | age, spherical equivalent at the beginning of the progression interval, sex, race, site, sex × age, race× age, autorefractor transition indicator, and  autorefractor transition indicator × site |  |  |
| Scheiman et al, COMET (2014)[40] | Y | 11 | Parents or children completed visual activity diary | Myopia -1.25 D ≦SER≦- 4.50D, for myopia stabilization status by age 15 |  | *All near work (Reading/Writing, Computer work/games and other near work.)* *(hrs/week)*  *Baseline near work (≦21 vs >21 hrs/week)*  *Total mean baseline near work (hrs/week)* | *OR (95% CI)for stable myopia by age 15*  *0.74 (0.43 to 1.29)*  *0.98 (0.96 to 1.00)* | baseline age, ethnicity | *stable: unstable=*  *21.2± 11.6: 24.6± 14.9* |  |

*Italic type: no statistical significance*

SER: spherical equivalent refractive error
